# Sonographic Measurement of Abdominal Esophageal Length as a Diagnostic Tool in Gastroesophageal Reflux Disease in Infants

**DOI:** 10.4103/1319-3767.74483

**Published:** 2011

**Authors:** Hamid Dehdashti, Masoud Dehdashtian, Fakher Rahim, Mehrdad Payvasteh

**Affiliations:** Radiology and MRI Department, Golestan Hospital, Ahwaz Jondishapour University of Medical Sciences, Ahwaz, Iran; 1 Physiology Research Center, Ahwaz Jondishapour University of Medical Sciences, Ahwaz, Iran; 2 Paediatric Surgery Ward, Imam Khomeini Hospital, Ahwaz Jondishapour University of Medical Sciences, Ahwaz, Iran

**Keywords:** Abdominal esophagus length, barium meal, GERD, sonography

## Abstract

**Background/Aim:**

This study was conducted to provide sonographic measurements of the abdominal esophagus length in neonates and infants with and without gastroesophageal reflux disease (GERD) and to investigate its diagnostic value. GERD severity was also evaluated and correlated with esophageal length. It is a prospective case-control study.

**Materials and Methods:**

This prospective case-control study comprised 235 neonates and infants (120 without reflux and 115 with reflux). There were 40 children without reflux in each of three age categories: less than 1 month, 1–6 months, and 6–12 months. Of the children with reflux, 40 were less than 1 month old; 37, 1–6 months; and 38, 6–12 months. The abdominal esophagus was measured from its entrance into the diaphragm to the base of gastric folds in fed infants. GERD was sonographically diagnosed and confirmed by a barium meal. The number of refluxes during a 10-min period were recorded.

**Results:**

Neonates and infants with reflux had a significantly shorter abdominal esophagus than subjects without reflux: the mean difference in neonates, 4.65 mm; 1–6 months, 4.57 mm; 6–12 months, 3.61 mm.

**Conclusions:**

Children with severe reflux had a shorter esophagus compared with those with mild and moderate reflux only in the neonate group. Therefore, thinking of GERD and carefully looking for its symptoms is necessary to avoid unnecessary utilization of healthcare resources in children with severe reflux.

Gastroesophageal reflux disease (GERD) can be defined as chronic symptoms or mucosal damage secondary to abnormal reflux of gastric contents into the esophagus.[1] According to Dent *et al*., the term GERD should be used to include all individuals who are exposed to the risk of physical complications from gastroesophageal reflux, or who experience clinically significant impairment of health-related well-being (quality of life) due to reflux-related symptoms, after adequate reassurance of the benign nature of their symptoms.[2,3] The severity of sign and symptoms of gastroesophageal reflux disease (GERD) in children varies according to age.[4,5] GERD is the most common esophageal disorder and one of the most frequent diseases of the gastrointestinal tract in children and infants.[6] GERD also is the most costly gastrointestinal disease in adults, and existing data suggest that treatment costs in children are as high as adults.[7]

GERD is a very common and usually benign physiological event in infants. A diagnosis of GERD is considered when gastroesophageal reflux is associated with presentations such as excessive irritability and crying, failure to thrive, feed refusal, apnea, and aspiration pneumonia. Many of these symptoms are not specific to GERD and can be due to other causes, such as feed intolerance, colic, constipation, or infection.[8–11] After excluding these possibilities, a trial of conservative measures, such as parental reassurance, upright positioning, feed thickeners, antacids, and elemental formulas may improve symptoms and obviate the need for pharmacologic therapy. A recent study of the efficacy of such measures showed a significant improvement in parent-reported symptoms in more than 50% of infants and normalization of symptom scores in 24% cases.[12]

Esophageal ultrasonography not only is non-invasive, readily available, repeatable and cheap, but also is a fast and highly sensitive technique[13–16] in the diagnosis of GERD in infants and children.[12–15] The esophageal ultrasonography studies in GERD have mainly focused on the evaluation of the gastroesophageal junction[17,18] and esophageal motility.[19–22] Sonographic GERD diagnosis was made by backward movement of gastric content into the esophagus and the visualization of the clearance of refluxate material.[23–25]

Literature suggests a low prevalence of GERD in Asia than in the West. In the USA, 20% of the population experiences the cardinal symptoms at least once a week.[26] The General Practice Research Database (GPRD) has been used to estimate an overall incidence of GERD in UK primary care of 4.5 new diagnoses per 1000 person-years. This extent has been reported to decrease to 4.8% and 2.5% in China.[26] The Asia-Pacific consensus report on the management of the GERD recognized that GERD is less common and milder in endoscopic survey in Asia than in the West and does not support the idea of increasing frequency of the disease.[27] A study from Iran on healthy blood donors, reported the prevalence of GERD as 14%.[28,29] Another study from Tehran reports daily heartburn and/or acid regurgitation in 2.1% and 4.7% of the university students and blood donors, respectively.[30] The aim of this study was to provide sonographic measurements of the abdominal esophagus length in neonates and infants with and without GERD and to investigate its diagnostic value.

## MATERIALS AND METHODS

### Patient’s selection and ultrasonography

A total of 235 children and infants <1 years age, 115 GERD and 120 non-affected as the control group were include in this study during time course from January 2006 to December 2008. The control group included infants without a clinical history of GERD, the absence of which was sonographically confirmed.

### Inclusion criteria

Patients with suspected GERD, symptomatic GERD, or endoscopically or histologically proven GERD, based on frequent vomiting or regurgitation, with at least one of the following: (a) poor weight gain or (b) irritability, excessive crying, or disturbed sleep that both the parent(s) and the doctor consider abnormal (but not due to colic) were included in this study.

### Exclusion criteria

All patients who used anti-acid drugs 24 h prior to sonography, with the presence of any systematic or metabolic diseases, history of any obstructive gastrointestinal disorders, and use of any drugs 24 h before sonography were excluded.

### Ultrasonography

Paediatric surgery specialists responsible for the management of patients admitted to the neonatal and infant wards of the Golestan Hospital contacted the study in collaboration with a sonography team when they encountered patients in the study.

Sonography was routinely carried out in all patients for diagnosing GERD in symptomatic children. Sonographically diagnosed GERD was confirmed by a barium meal. Esophageal length was measured carefully from the point at which it penetrated the diaphragm to the base of the triangular pad of gastric folds at the anterior surface of the fundus of the stomach. Triangular pad, representing the radiation away from the cardiac orifice, was considered the point of entrance of esophagus into the stomach. The sonographic measurements of the abdominal esophagus length were undertaken, and the mean value was considered. The GERD was divided into three groups based on the number of refluxes in 10 min time interval. The groups include (1) mild, less than three refluxes in 10 min; (2) moderate, four to six refluxes in 10 min; (3) severe, more than six refluxes in 10 min.

During the test, children were allowed unrestricted diet and activity. Patients with known history of gastric ulcer or who resist the sonography were excluded from the study. Informed consent was obtained from the patients and their parents, and patient anonymity was preserved. The research protocol had been approved by Local Ethics Committee of Ahwaz Jondishapour University of Medical Sciences. The patients and control groups were also divided into three groups according to age include, group 1, less than 1 month; group 2, 1 to 6 months; and group 3, 6 to 12 months of age. During sonography, the patients were awake while they were relaxed in rest position. After using sufficient fluid according to patient’s age, such as milk or water, patients were studied in supine position using a color ultrasound machine with a 7.5 MHz linear array transducer with a color flow-mapping capability (Esaote Biomedica AU3, Italy). The stomach and lower segment of esophagus were studied.

### Statistical analysis

Percentages were used for categorical data, whereas continuous numerical data were expressed as mean ± standard deviation. The results are given in their 95% confidence intervals. Univariate analysis was performed by using the independent samples t-test and ANOVA followed by Kruskal-Wallis and Bonferroni-Dunn *post hoc* tests whenever appropriate. *P*< 0.05 indicated statistical significance. Statistical interpretation of data was performed using the SPSS software for windows version 13 (SPSS Inc., Chicago, IL, USA).

## RESULTS

**Figure 1:**
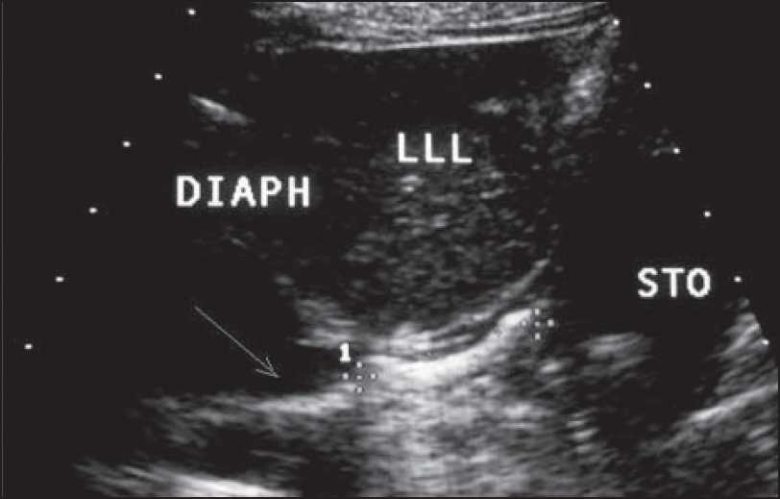
Retrograde flow of gastric contents through the lower esophagus detected by sonography (arrow)

**Figure 2:**
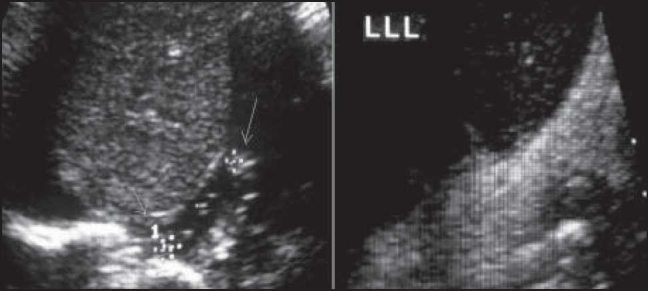
Measurement of the intra-abdominal portion of esophagus using left liver lobe as an ultrasonic window

A total of 235 children with a mean age of 3.5 and 4.1 months in patients and control groups, respectively, were entered in the study (*P*>0.05). Among the patients, the male to female ratio was 2.2 (73:32) in the patient group and 0.93 (58:62) in the control group. The most common symptom in patients was vomiting seen in 41 patients (35.65%). Other signs and symptoms are mentioned in Table 1. There was retrograde flow of gastric contents through the lower esophagus detected by sonography [Figure 1]. We have measured the intra-abdominal portion of esophagus using left liver lobe as an ultrasonic window which is shown in [Figure 2].

**Table 1:** Presenting symptoms in the 115 infants and children admitted for suspected GERD

Signs and symptoms	Number (%)
Vomiting	41 (35.65)
Failure to thrive	29 (25.22)
Weight loss	14 (12.18)
Hematemesis	13 (11.3)
Recurrent pneumonia	5 (4.35)
Recurrent wheezing	4 (3.48)
Chronic cough	3 (2.6)
Apneic spells	2 (1.74)
Iron defi ciency anemia	2 (1.74)
Epigastria pain	1 (0.87)
Irritability	1 (0.87)
Total	115 (100)

In the sonographic evaluation, the severity of the disease is mentioned as mild in 36 (31.6%), moderate in 56 (49.2%), and severe in 23 (19.2%) patients. There was a significant difference in the mean of sub-diaphragmatic esophageal length in all three groups of patients compared to control Table 2. For differentiation of patients according to severity of reflux (three mentioned groups), we compared each variable with others individually. Finally statistical analysis showed a significant difference for all pair wise comparisons of these variables for differentiation of reflux’s severity in patients Table 3.

**Table 2:** Comparing the sub diaphragmatic esophageal length (mm) between patients and control groups

Study groups		No.	Mean ± SD	Range	95% CI	*P* value
Group 1	Patients	40	17.36 ± 1.16	15 - 19	16.98 – 17.73	<0.001
	Control	40	21.95 ± 1.83	21 - 24	21.36 – 22.53	
Group 2	Patients	40	20.93 ± 1.63	18 - 23	20.34 – 21.52	<0.001
	Control	37	25.47 ± 0.89	24 - 27	25.19 – 25.76	
Group 3	Patients	40	23.36 ± 3.57	22 - 26	22.13 – 24.60	<0.05
	Control	38	26.97 ± 1.31	25 - 29	26.55 – 27.39	
Total	Patients	120	20.47 ± 3.54	15 - 26	16.93 – 24.53	<0.05
	Control	115	24.81 ± 2.53	21 - 29	21.6 – 27.87	

Group 1: less than 1 month; group 2: 1 to 6 months; and group 3: 6 to 12 months of age

**Table 3:** Comparing the sub diaphragmatic esophageal length (mm) between severity group in patients and group

Study groups		No.	Mean ± SD	95% CI	Groups/*P* value
Group 1	Mild	20	18.31 ± 0.65	17.96 – 18.66	Mi – Mo / <0.001
	Moderate	12	17.15 ± 0.85	16.70 – 17.60	Mo – Se / <0.001
	Severe	8	15.80 ± 0.64	21.36 – 22.53	Se – Mi / <0.001
	Total	40	17.63 ± 1.16	16.98 – 17.73	-
Group 2	Mild	19	22.25 ± 0.71	21.73 – 22.76	Mi – Mo / <0.001
	Moderate	15	20.63 ± 1.5	19.90 – 21.35	Mo – Se / <0.05
	Severe	3	18.5 ± 0.50	17.25 – 19.74	Se – Mi / <0.05
	Total	37	20.93 ± 1.63	20.34 – 21.52	-
Group 1	Mild	19	24.96 ± 0.71	24.58 – 25.35	Mi – Mo / <0.001
	Moderate	12	23.46 ± 0.78	23.02 ± 23.90	Mo – Se / <0.05
	Severe	7	19.5 ± 7.73	12.34 – 26.65	Se – Mi / <0.05
	Total	38	23.36 ± 3.75	22.13 – 24.60	-

Group 1: less than 1 month; group 2: 1 to 6 months; and group 3: 6 to 12 months of age; Mild (Mi), less than three refl uxes in 10 min; moderate (Mo), four to six refl uxes in 10 min; severe (Se), more than six refl uxes in 10 min.

## DISCUSSION

GERD occurs during the lifetime of most people especially in the childhood and newborn period. Different methods have been introduced for diagnosis of the disease during childhood including barium meal, PH monitoring, manometry, and sonography.[30–32] Sonographic detection of GERD is mainly based on the detection of the returning gastric fluid to esophagus, so the Doppler study has increased the sensitivity of the mentioned method.[32] In agreement with our findings, Koumanidu *et al*.[32] compared abdominal esophagus length of 150 healthy to 108 GERD neonates and infants. They showed that neonates and infants with reflux had a significantly shorter abdominal esophagus than subjects without reflux: the mean difference in neonates, 4.8 mm; 1-6 months, 4.5 mm; 6-12 months, 3.4 mm, while in our study their values were in neonates, 4.65 mm; 1-6 months, 4.57 mm; 6-12 months, 3.61 mm. Both studies showed that the children with severe reflux had a shorter esophagus compared with those with mild and moderate reflux only in the neonate group.

In healthy infants and children, abdominal esophagus length has been sonographically measured by two different groups of researchers previously.[33,34] However, in pediatric patients with GERD, sonographic measurements of abdominal esophagus length have been undertaken in a few studies.[32] In our study, abdominal esophagus length was measured in children having GERD to confirm the results of other studies suggesting that with an inadequate length of the abdominal esophagus, the prevalence of GERD is high.[35–37] When discriminating between mild, moderate, and severe GERD on the basis of the number of refluxes per 10 min, we found that only neonates with severe reflux had a significantly shorter abdominal esophagus compared with neonates with mild reflux. Such differences related to GERD severity were found either between other category groups of GER severity in this age group or between any pair comparison in older infants. In contrast, Jang *et al*.[8] found no significant correlation between increased number of refluxes and severity of GER when comparing findings of color Doppler sonography and pH measurements in children of 2 months to 10 years old.

In conclusion, the results of our study suggest sonography as a single and adequate diagnostic test for GERD. The measurement of the abdominal esophagus length confirms sonographic diagnosis of GERD, which until now was based solely on passage of gastric content into the proximal esophagus during a 10-min period. Therefore, thinking of GERD and carefully looking for its symptoms is necessary to avoid unnecessary utilization of healthcare resources in children with severe reflux.
